# Exploring the regulatory mechanism of intestinal flora based on PD-1 receptor/ligand targeted cancer immunotherapy

**DOI:** 10.3389/fimmu.2024.1359029

**Published:** 2024-03-28

**Authors:** Xinran Gao, Jingting Jiang

**Affiliations:** ^1^ Department of Tumor Biological Treatment, The Third Affiliated Hospital of Soochow University, Changzhou, Jiangsu, China; ^2^ Jiangsu Engineering Research Center for Tumor lmmunotherapy, The Third Affiliated Hospital of Soochow University, Changzhou, Jiangsu, China; ^3^ Institute of Cell Therapy, The Third Affiliated Hospital of Soochow University, Changzhou, Jiangsu, China

**Keywords:** PD-1, anti-PD-1, intestinal flora, ICI, immunotherapy

## Abstract

Serving as a pivotal immunotherapeutic approach against tumors, anti-PD-1/PD-L1 therapy amplifies the immune cells’ capability to eliminate tumors by obstructing the interaction between PD-1 and PD-L1. Research indicates that immune checkpoint inhibitors are effective when a patient’s gut harbors unique beneficial bacteria. As such, it has further been revealed that the gut microbiome influences tumor development and the efficacy of cancer treatments, with metabolites produced by the microbiome playing a regulatory role in the antitumor efficacy of Immune checkpoint inhibitors(ICBs). This article discusses the mechanism of anti-PD-1 immunotherapy and the role of intestinal flora in immune regulation. This review focuses on the modulation of intestinal flora in the context of PD-1 immunotherapy, which may offer a new avenue for combination therapy in tumor immunotherapy.

## 1 Introduction

The treatment strategies for tumors are combination of multiple therapies, including surgery, chemotherapy, radiotherapy, and immunotherapy (1). Recently, researchers have paid more attention to immunotherapy, making immune checkpoint inhibitors an important treatment for most types of tumors (2).

Extensive research has delved into the effectiveness of the PD-1/PD-L1 pathway in cancer. Anti-PD-1 treatment hinders the liberation of negative modulators in the immune checkpoint, a function with broad applicability across malignancies, offering a sustained response in contrast to conventional therapies (3). PD-1 is a widely studied inhibitory regulatory receptor, indispensable in maintaining immune system homeostasis and regulating the function of T cells. Although anti-PD-1/PD-L1 therapy has demonstrated its effectiveness against tumors in certain patients, its efficacy is not universal, encountering challenges related to treatment resistance in some cases (4). In fact, it may also be necessary to regulate cancer immune checkpoints, promote immune tolerance to those immune checkpoints, and control abnormal angiogenesis, among other factors (5). Therefore, a combination treatment strategy is considered a reasonable and feasible way to achieve the most efficacious therapeutic effect.

Intestinal flora can influence the therapeutic effectiveness of various cancer treatments, including chemotherapy, radiation, and immunotherapy (6). Currently, there is widespread concern about whether the gut microbiome can modify the innate immune response to tumors and influence the effectiveness of immunotherapy (7). Studies have uncovered that the gut microbiome has the capacity to impact both tumor progression and the effectiveness of cancer treatments (8). The success of ICB therapy hinges on the existence of particular beneficial bacteria in the patient’s gut, and certain microbial metabolites have been identified as regulators of ICB efficacy. This review focuses on the regulation of intestinal flora against PD-1 immunotherapy, which may provide a new avenue for combination therapy in tumor immunotherapy.

## 2 Results

### 2.1 Specific elucidation and research progress of anti-PD-1 mechanism

#### 2.1.1 The typical pathways of anti-PD-1 therapy

Programmed cell death protein 1 (PD-1) is a transmembrane protein of type I, encoded by the PDCD1 gene (9). Its primary expression occurs on activated T cells (CD4^+^ T cells, CD8^+^ T cells). However, it is also found on B cells, macrophages, monocytes, natural killer T cells, and dendritic cells (DCs). PD-1 stands out as one of the extensively studied inhibitory regulatory receptors, functioning as a crucial immune checkpoint that governs T cell and B cell antigen responses. Its role is indispensable in precisely modulating T cell function and preserving the balance of the immune system (9). The interaction between PD-1 and its ligand programmed cell death protein 1 ligand 1(PD-L1) can lead to T-cell exhaustion and the suppression T-cell function (10).

**Figure 1:**
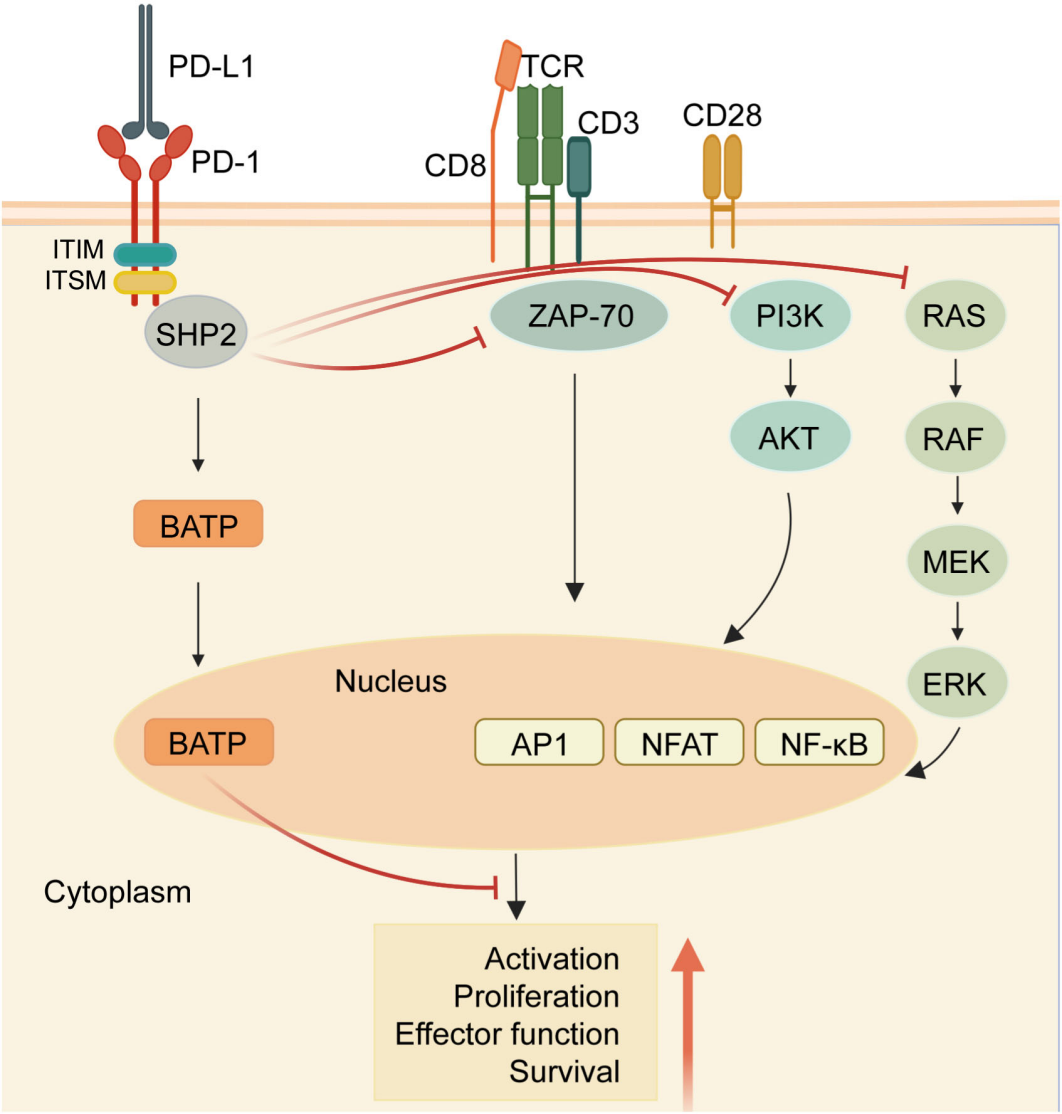
PD-1/PD-L1 signaling in T cells. When the receptor PD-1 binds to its ligand PD-L1, the PD-1 ITSM is phosphorylated at tyrosine residues, leading to the recruitment of protein tyrosine phosphatases (PTPs) such as SHP2. SHP2 dephosphorylates ZAP70 and antagonizes positive signals through the TCR and CD28 receptors, thereby inhibiting T cell function and T cell exhaustion. In addition, PD-1 can inhibit T cell function by up-regulating the expression of the transcription factor BATF. (Created with BioRender.com).

Within the cytoplasmic domain of PD-1, two tyrosine residues are present: an immunoreceptor tyrosine inhibitory motif (ITIM) and an immunoreceptor tyrosine switching motif (ITSM) (11). Upon binding to a ligand, primarily PD-L1, the tyrosine residues situated in the PD-1 ITSM undergo phosphorylation, recruiting protein tyrosine phosphatases (PTPs). These PTPs antagonize the signals generated by T cell receptor (TCR) and CD28, inhibiting the effect of TCR activation (12). For example, shp2 inhibits the RAS-ERK pathway, and ZAP-70, the PI3K-AKT pathway. Therefore, PD-1 binding to PD-L1 reduces transcription factor activation, resulting in the inhibition of proliferation, activation, and cytokine production in T cells (**Figure 1**). This culminates in the inhibition of metabolic alterations and the impairment of cytotoxic T lymphocytes (CTLs) killing function, ultimately leading to the demise of activated T cells (13). The PD-L1 signaling pathway is implicated in various cell types, encompassing dendritic cells, macrophages, T cells, B cells, and mast cells. Beyond hematopoietic cells, nonhematopoietic cells such as vascular endothelium, islet cells, placental trophoblasts, and keratinocytes are also involved. In healthy tissues, the predominant mechanism for maintaining physiological peripheral immune tolerance involves the elevated expression of PD-L1. This control mechanism mitigates tissue autoimmunity following prolonged inflammatory responses to tissue injury. However, heightened PD-L1 expression diminishes T-cell-mediated cytotoxicity, facilitating evasion from the immune system (14).Tumor-infiltrating lymphocytes (TILs) stand out as the principal effector cells within the tumor tissue microenvironment, expressing PD-1 (15). Throughout tumor progression, tumor cells express negative checkpoint regulators that stifle immunity, allowing them to elude immune surveillance (16). Furthermore, interactions involving anti-PD-1/PD-L1 also contribute to the restoration of CD8^+^ T cell function within tumors (17).

Crucially, the inhibition of the PD-1/PD-L1 pathway instigates the activation of tumor-specific cytotoxic T cells within the tumor microenvironment (15). The coalescence of PD-L1 and PD-1 fosters the induction of interleukin-10 and T cell apoptosis, leading to inactivation and depletion of T cells (15, 18). Additionally, PD-L1 serves as a protective “shield” for tumor cells against the cytotoxic response mediated by CD8^+^ T cells (19). Furthermore, CD80, expressed on activated T cells and antigen-presenting cells (APCs), acts as a receptor for PD-L1, transmitting inhibitory signals upon binding to PD-L1 (18). Moreover, PD-L1 can function as a receptor, transmitting signals from T cells to tumor cells, resulting in resistance of tumor cells to lysis. Consequently, the blockade of PD-1 and PD-L1 interaction effectively enhances the cytotoxicity of tumor-infiltrating lymphocytes(TILs) against tumor cells (20).

#### 2.1.2 Progress in anti-PD-1 research and its combined therapy

The effectiveness of the PD-1/PD-L1 pathway has undergone thorough examination in the realm of cancer. Anti-PD-1 treatment, by impeding the release of negative modulators within the immune checkpoint, exhibits functionality with broad relevance across malignancies, yielding a sustained response when contrasted with conventional therapies (3). Employing the blockade of the PD-1/PD-L1 checkpoint has become the standard therapeutic approach for various cancers. Moreover, the inhibition of PD-1/PD-L1 is currently under intense scrutiny in clinical trials for the treatment of numerous other diseases (21). Based on clinical trial results, antibodies against PD-1 and PD-L1 are currently being used to treat multiple cancers (**Table 1**).

**Table 1:** Monoclonal antibodies against PD-1 approved by the US Food and Drug Administration.

Name of antibody	Mechanisms of action of antibodies	Types of Antibodies	Affected types of cancer	Reference
Atezolizumab (Tecentriq, Genetech/Roche)	● Bound to the ligand PD-L1 on tumor cells and blocked the binding of PD-L1 to its inhibitory receptor PD-1 by	Anti-PD-1 humanized IgG1 antibody	Metastatic NSCLC and Advanced or Metastatic UC	(22)
Avelumab	● Inhibited the interaction of PD-1/PD-L1	Anti-PD-1 humanized IgG1 antibody	MCC and UC	(23)
Camrelizumab (SHR-1210)	● Blocked the interaction of PD-1 with its ligands ● Dampened tumor cells’ immune evasion	Anti-PD-1 humanized IgG4 monoclonal antibody	Relapsed or refractory cHL, B cell lymphoma, oesophageal squamous cell carcinoma, gastric/gastroesophageal junction cancer, HCC, Nasopharyngeal Cancer(NPC), NSCLC.	(24, 25)
Cemiplimab	● Blocked T-cell inactivation.	Anti-PD-1 humanized monoclonal antibody	Basal cell carcinoma (BCC), NSCLC, Squamous Cell Carcinoma (SCC)	(26)
Dostarlimab	● Bound to PD-1 on T cells and prevented PD-1 from interacting with its ligands, activating the immune response	Anti-PD-1 humanized IgG4 antibody	Endometrial Cancer	(27)
Durvalumab(Imfinzi, AstraZeneca)	● Inhibited PD-L1 interactions with PD-1 and CD80	Anti-PD-1 humanized IgG1 antibody	Advanced UC and NSCLC	(28)
Nivolumab	● Blocked the interaction of PD-1 with PD-L1 and PD-L2 by binding to the PD-1 receptor ● Inhibited T-cell apoptosis, inactivation, and exhaustion ● Promoted interleukin-10 expression due to PD-L1 and PD-1 binding ● Preserved T-cell function	Anti-PD-1 human monoclonal antibody,	NSCLC, melanoma, advanced RCC, classical Hodgkin lymphoma (cHL), CRC and HCC, Squamous Cell Carcinoma of the Head and Neck (SCCHN), UC	(29–32)
Pembrolizumab(Keytruda, Merck & Co/MSD)	● Blocked the PD-1’s binding to its immunosuppressive ligand ● Attenuated the inhibition of T cells	Anti-PD-1 humanized IgG4 monoclonal antibody	Melanomas, NSCLC, SCCHN, cHL, UC, primary mediastinal large B-cell lymphoma(PMBCL), advanced cervical cancer, HCC and Metastatic Merkel cell carcinoma (MCC), advanced or metastatic Gastroesophageal Junction Adenocarcinoma	(33–35)
Prolgolimab (formerly BCD-100)	● Contained the Fc-silencing L234A/L235A (LALA) mutation by eliminating the interaction between the Fc region of the antibody and FcγR expressed on various immune cells. ● Blocked the effector function of antibodies and enhancing immunity.	Anti-PD-1 humanized IgG1 antibody	Advanced Melanoma	(36)
Sintilimab	● Blocked the interaction of PD-1 with its ligands ● Recovered T-cell antitumor efficacy	Anti-PD-1 humanized IgG4 antibody	Hodgkin lymphoma(HL), natural killer/T cell lymphoma cancer and advanced NSCLC.	(37)
Tislelizumab	● Inhibited the binding of PD-1 to FcγR on macrophages	Anti-PD-1 humanized IgG4 antibody	Metastatic PD-L1 UC and cHL	(38)
Toripalimab	● Bound to PD-1 and blocked the interaction with its ligands	Anti-PD-1 humanized monoclonal antibody	Lung Cancer, Melanoma, Neuroendocrine Tumors, NPC and Digestive Tract, Hepatobiliary, Pancreatic Tumors.	(39)
Zimberelimab	● Bound to PD-1 and blocked the interaction with its ligands	Anti-PD-1 humanized monoclonal antibody	NSCLC, and relapsed or refractory cHL, Cervical Cancer	(40)

Despite the robust anti-tumor effects demonstrated in certain patients by α-PD-1/PD-L1 therapy, the majority are unable to derive sole benefits from this treatment, owing to the existence of various primary and secondary immune escape mechanisms (4). In fact, it may also be necessary to regulate cancer immune checkpoints and promote immune tolerance towards those immune checkpoints, as well as control abnormal angiogenesis, and so on (5). Eliminating these negative factors could raise the efficacy and mitigate drug resistance. In turn, reinforcing positive factors could improve the response to anti-PD-1/PD-L1 medical treatment (41). Therefore, a combination treatment strategy is considered to be a reasonable and feasible way to achieve the most efficacious therapeutic effect. It has been identified that some combination therapies can work synergistically with anti-PD-1/PD-L1 by raising the antigen release, and/or APC function and activity (42).

Studies have uncovered that the microbiome residing in the gut has the capacity to impact both tumor development and the effectiveness of cancer treatment (8). The success of ICB therapy hinges on the existence of particular beneficial bacteria in the patient’s gut, and certain microbial metabolites have been identified as regulators of the efficacy of ICBs. When dietary intake is degraded by the gut microbiome, various metabolites are produced, and liquid chromatography-tandem mass spectrometry can accurately screen the metabolites of intestinal flora (43). Some of these metabolites can trigger an inflammatory response, which aids the regulation of immune function (44). The presence of specific intestinal flora can cause an increase in the efficacy of PD-1 drugs four-fold. For example, certain bacteria (*Lactobacillus johnsonii*, *Bifidobacterium pseudolongum*, *Olsenella species*) produce inosine. In combination with checkpoint blockade immunotherapy, inosine can increase its effectiveness, promote the anti-tumor capacity of T cells, and ultimately inhibit tumor development (44). In summary, a deeper understanding of the interactions between the gut microbiome and the host immune system is of great importance for developing new cancer treatment strategies.

### 2.2 Gut microbiota and anti-tumor immunity

Tumor and gut microbiota determine cancer progression and response to treatment through their effects on innate and adaptive immunity (45). Intestinal flora can directly affect the progression and metastasis of tumors. Consequently, the presence and advancement of intestinal tumors stimulate the activity of intestinal flora. The interaction between tumors and gut microbiota plays a pivotal role in influencing cancer progression and the response to treatment, exerting effects on both innate and adaptive immunity (45). Intestinal flora can directly affect the progression and metastasis of tumors. As such, intestinal flora are stimulated by the occurrence and development of intestinal tumors.

#### 2.2.1 Gut microbiota regulates innate immunity

The gut microbiota modulates innate immunity by regulating various immune cells, such as dendritic cells (DCs), monocytes, and natural killer (NK) cells.

The gut microbiota plays a regulatory role in DCs, which constitute a specialized group of antigen-presenting cells crucial for T cell activation and anti-tumor immunity (46). When *Bifidobacterium* is orally administered, it fosters the activation of DCs, consequently enhancing tumor-specific CD8^+^ T cell responses (47). Additionally, *Bacteroides fragilis* can promote the maturation of DCs and augment the IL-12-dependent Th1 cell immune response, thereby amplifying the anti-tumor efficacy of anti-CTLA-4 treatment (48).

Moreover, the gut microbiome exerts control over monocytes. Monocolonization with cdAMP-producing *Akkermansia muciniphila*(*AKK*) triggers a cascade involving monocytes, IFN-I, NK cells, and DCs, ultimately reinforcing anti-tumor responses (49). Similarly, *Bifidobacterium*, through STING signaling, supports CD47-based immunotherapy (50). Furthermore, *Bacteroides fragilis* is implicated in the up-regulation of the proportion of M1 macrophages, enhancing the expression of CD80 and CD86 on the cell surface and thus promoting innate immunity (51).

The gut microbiota plays a role in regulating NK cells, which influence anti-tumor immunity by modulating the abundance of DCs and CD8^+^ T cells in the TME (46). *Lactobacillus plantarum* has been shown to elevate NK cell activity by up-regulating the expression of natural cytotoxic receptor (NCR) protein (52). Furthermore, the intratumoral presence of *Bifidobacterium* and the subsequent heightened activation of NK cells have been demonstrated to induce anti-tumor immunity (53).

#### 2.2.2 Intestinal microbiota regulates adaptive immunity

The gut microbiota exerts control over CD8^+^ T cells. In a melanoma clinical trial, a high abundance of *Clostridiales*, *Ruminococcaceae*, or *Faecalibacterium* is linked to increased antigen presentation. In comparison to patients with low abundance, this results in enhanced functionality of CD4^+^ and CD8^+^ T cells in both peripheral blood and the TME. Such enhancement is advantageous for the anti-tumor effectiveness of immune checkpoint inhibitors (ICI) (8). Another clinical trial provides evidence that the relative abundance of *Enterococcus* increases in patients’ tumors after receiving fecal microbiota transplantation (FMT) and anti-PD-1 treatment. This increase correlates with intratumoral CD8^+^ T cell infiltration and tumor cell necrosis (54). Furthermore, *Bifidobacterium* has been shown to increase the abundance of CD8^+^ T cells and enhance the efficacy of ICI treatment (47).

The gut microbiome exercises control over CD4^+^ T cells. The colonization of *segmented filamentous bacteria* (*SFB*) is particularly effective in inducing the growth of Th17 cells within the small intestine (55). Th17 cells, a vital component of CD4^+^ effector T cells, are proficient producers of IL-17, which plays a crucial role in host defense against extracellular pathogens (56). *SFB* induces small intestinal epithelial cells to produce serum amyloid A (SAA), which then activates CX3CR1^+^ phagocytes to produce IL-1β and IL-23. The synergistic action of IL-1β and IL-23 promotes the production of IL-22 in group 3 innate lymphoid cells (ILC3). Subsequently, IL-22 positively influences phagocytes, mediating IL-1β production and ultimately elevating IL-17 production in RORγt^+^ CD4^+^ T cells (55).Studies have shown that gut microbes exert control over the functionality of Treg cells by producing metabolites, including short-chain fatty acids (SCFAs), providing protection against colitis in mice through a mechanism dependent on Ffar2 (GPR43) (57). The genus *Clostridium*, especially *Clostridium leptum* and *Clostridium coccoides*, demonstrates the ability to induce Treg activity in both humans and mice (58). Moreover, SCFAs, particularly butyrate, possess the capability to augment the acetylation of Foxp3 sites in Tregs (59).

#### 2.2.3 Regulatory mechanisms of immune cells by gut microbiota metabolites

Microbiota metabolism generates a multitude of metabolites, serving as crucial signaling factors and energy substrates that influence intestinal tumors (60). The key metabolites of gut microbiota encompass inosine, short-chain fatty acids (SCFAs), and anacardic acid, among others.

Inosine, a purine metabolite derived from gut microbes *AKK* and *Bifidobacterium pseudolongum* (*B. pseudolongum*), exhibits synergistic antitumor effects when combined with ICI therapy (44). Firstly, the treatment of tumor cells with inosine enhances the activation of IFN-γ and TNFα signaling pathways. IFN-γ, in turn, boosts the release of perforin and granzyme, augmenting antigen presentation and ultimately fostering anti-tumor immunity (61). Secondly, inosine enhances the efficacy of ICI by influencing the adenosine 2A receptor (A2AR) on T lymphocytes (44). Lastly, inosine can also serve as an alternative carbon source, providing energy for CD8^+^ T cells (62).

SCFAs, metabolites of gut microbiota, are also implicated in cancer antibody therapy (63). Firstly, *Faecalibaculum rodentium PB1* and *Helicia biformis* (*H. biformis*) metabolize butyrate, which inhibits histone deacetylase (HDAC) and activates the NFATc3 transcription factor, leading to the inhibition of tumor cells (64). Secondly, SCFAs enhance anti-tumor immune responses. Butyrate, for instance, upregulates the expression of inhibiting DNA binding 2 (ID2) in CD8^+^ T cells, thereby augmenting the anti-tumor cytotoxicity of CD8^+^ T cells (65). Finally, SCFAs can serve as an energy source for anti-tumor immune cells by modulating metabolic pathways such as glycolysis, the tricarboxylic acid (TCA) cycle, and β-oxidation (66).

In addition to purines and SCFAs, several studies have verified that anacardic acid can also boost antitumor immunity. Anacardic acid activates innate immunity by phosphorylating mitogen-activated protein kinases (MAPKs), initiating the classical activation pathway of macrophages (67). Furthermore, in certain breast cancer models, it has been confirmed that anacardic acid elevates the levels of tumor-infiltrating NK cells and CTLs, inducing apoptosis in tumor cells (68).

Additionally, certain gut microbiota metabolites have been identified to dampen antitumor immune responses. For instance, some gut microbes generate the bile acid metabolite 3-oxolithocholic acid, which hinders the differentiation of Th17 cells (69). Moreover, the secondary bile acid 3β-hydroxydeoxycholic acid has been found to attenuate the immune activity of DCs, increase the number of Tregs, and facilitate immune escape (70).

These discoveries provide critical insights into regulatory mechanisms of immune cells by gut microbial metabolites, offering potential directions for developing novel anti-tumor therapies in the future.

#### 2.2.4 Relationship between the gut microbiota and irAEs

The relationship between gut microbiota and immune-related adverse events (irAEs) triggered by immune checkpoint inhibitors (ICIs) is increasingly gaining attention. A specific category of toxicities resulting from the immune system’s hyperactivation induced by ICIs is termed irAEs (71). The gut microbiota is linked to the occurrence, type, and severity of these irAEs. This connection is particularly evident in cases of immune-related colitis, where it is speculated to operate through the regulation of metabolic pathways (72).

In a research endeavor involving 93 fecal samples obtained from 37 cancer patients undergoing anti-PD-1 treatment, individuals experiencing irAEs exhibit lower levels of *Bifidobacterium*, *Faecalibacterium*, and *Agathobacter*. Conversely, the abundance of *Erysipelatoclostridium* is notably higher in these patients. Those with colitis-type irAEs showcase reduced abundance of *Bacteroides* and *Bifidobacterium*, while *Enterococcus* has a higher prevalence (72). A thorough examination of the gut microbiota in 150 patients post-ICI treatment reveals that those with severe irAEs display elevated levels of *Streptococcus*, *Paecalibacterium*, and *Stenotrophomonas*. In contrast, patients with mild irAEs exhibit heightened levels of *Faecalibacterium* and unidentified *Lachnospiraceae (* 73).

Analysis of gut microbiota in patients after ICI therapy has shown significant differences in the abundance of certain microbial communities between severe and mild irAEs, indicating that the diversity and abundance of gut microbiota may serve as important predictive indicators for the occurrence of irAEs (74). Particularly in patients with ICI-related colitis, changes in the gut microbiota are closely associated with disease progression (75). These findings highlight the potential role of gut microbiota in ICI therapy and offer a new perspective for a better understanding of the mechanisms, prevention, and treatment of irAEs.

### 2.3 Regulation of intestinal flora in anti-PD-1 immunotherapy

The effectiveness of utilizing ICBs to augment immunotherapy has been documented to diminish in the presence of antibiotics, while a heightened effectiveness has been noted in the presence of specific gut microbiota (76). The manipulation of gut microbiota composition has been shown to enhance the effectiveness of anti-PD-1/PD-L1 therapies (77). Numerous clinical studies have reported that FMT can regulate the intestinal microbiota in patients and effectively mitigating resistance to anti-PD-1/PD-L1 treatment in melanoma (78). The presence of an abundance of *Clostridium* or *Difilibacterium* in the gut of melanoma patients indicates a favorable response to anti-PD-1 therapy (8) (**Table 2**).

**Table 2:** Regulation of intestinal flora against PD-1 immunotherapy.

Intestinal flora	Effects on immunity	Effects on anti-PD-1 therapy	Affected types of cancer	Reference
*Akkermansia muciniphila*	● Increased number CXCR3^+^CCR9^+^CD4^+^T cells2. ● Improved DC capability and IL12 production Increased IFN-γ ● production of memory T cells	Enhanced PD-1 blocking effect	RCC, Melanoma, and NSCLC	(76)
*Alistipes indistinctus*	● Reduced resistance to anti-PD-1	Enhanced PD-1 blocking effect	RCC, Melanoma, and NSCLC	(76)
*Bacteroidales*	● Upregulation of MDSC and Tregs	Reduced PD-1 blockade effect	Melanoma, and NSCLC	(8)
*Bifidobacterium*	● Enhanced DC activity Up-regulated ● tumor-specific CD8^+^ T cells ● Up-regulated proinflammatory cytokines	Enhanced PD-1 blocking effect	CRC, BC, Melanoma, NSCLC, and RCC	(47)
*Bifidobacterium adolescentis* *Bifidobacterium longum*	● Increased IFN-γ secretion ● Increased CD8^+^ T cell ● tumor infiltration ● Induction of DC pair maturation, promotion of cytokine secretion	Enhanced PD-1 blocking effect	Melanoma	(79)
*Collinsella aerofaciens* *Klebsiella pneumonia* *Parabacteroides merdae* *Veillonella parvula*	● Reduced peripherally induced Tregs	Enhanced PD-1 blocking effect	Melanoma	(79)
*Enterococcus faecium*	● The metabolite SagA induces the release of peptidoglycan fragments to produce NOD2 active polypeptides, reducing peripherally induced Tregs	Enhanced PD-1 blocking effect	Melanoma	(80)
*Enterococcus hirae*	● Induces CD8^+^ T cells Inhibition of IL-6, TGF-β, IL-17 expression	Enhanced PD-1 blocking effect	Melanoma, HepG-2 Cancer and HT-29 Human Colon Cancer	(44)
*Faecalibacterium*	● Increased CD4^+^, CD8^+^ T cells in the tumor	Enhanced PD-1 blocking effect	Melanoma	(8)
*Faecalibacterium prausnitzii*	● Increased CTL concentrations in the tumor microenvironment	Enhanced PD-1 blocking effect	Melanoma	(81)
*Lactobacillus johnsonii*	● Produces the metabolite hypoxanthine, improves the effect of immunotherapy	Enhanced PD-1 blocking effect	Colon Cancer	(44)
*Lactobacillus rhamnosus GG*	● Increased T-cell and DC infiltration of tumor cells ● Induces the production of type I interferon (IFN) and improves the production of anti-tumor CD8^+^ T cells ● Induces IFN-β signalling by cGAS/STING/TANK binding IFN regulatory factor 7 axis in DCs, promotes immune cell activation	Enhanced PD-1 blocking effect	CRC and Melanoma	(82)
*Olsenella*	● Increased secretion of IL-12 and IFN-γ ● Produces the metabolite inosine, enhances the anti-tumor efficacy of T cells	Enhanced PD-1 blocking effect	CRC, BC, Melanoma, NSCLC	(44, 80)
*Roseburia intestinalis* *Ruminococcus obeum*	● Enriched in patients with anti-PD-1 resistance	Reduced PD-1 blockade effect	Melanoma	(79)
*Ruminococcaceae*	● Increased numbers of effector T cells in peripheral blood and TIL ● CD8^+^/FOXP3^+^CD4^+^T cell activity in the tumor microenvironment ● Increased CD8^+^ T cell infiltration in the tumor Potentiated effects of ● CD4^+^ and CD8^+^ T cells Improved	Enhanced PD-1 blocking effect	Melanoma, and NSCLC	(8)

#### 2.3.1 Enhancing the impact of intestinal flora on anti-PD-1 immunotherapy

Anti-PD-1/PD-L1 treatment inhibits the negative signaling of the PD-1 intracellular domain (ITIM, ITSM) transduction (11). Biomarkers highly correlated with anti-PD-1/PD-L1 therapeutic efficacy include PD-L1 expression levels and TILs status (83). Crucially, the gut microbiome plays a vital role in influencing the effectiveness of immunotherapy (84).

**Figure 2:**
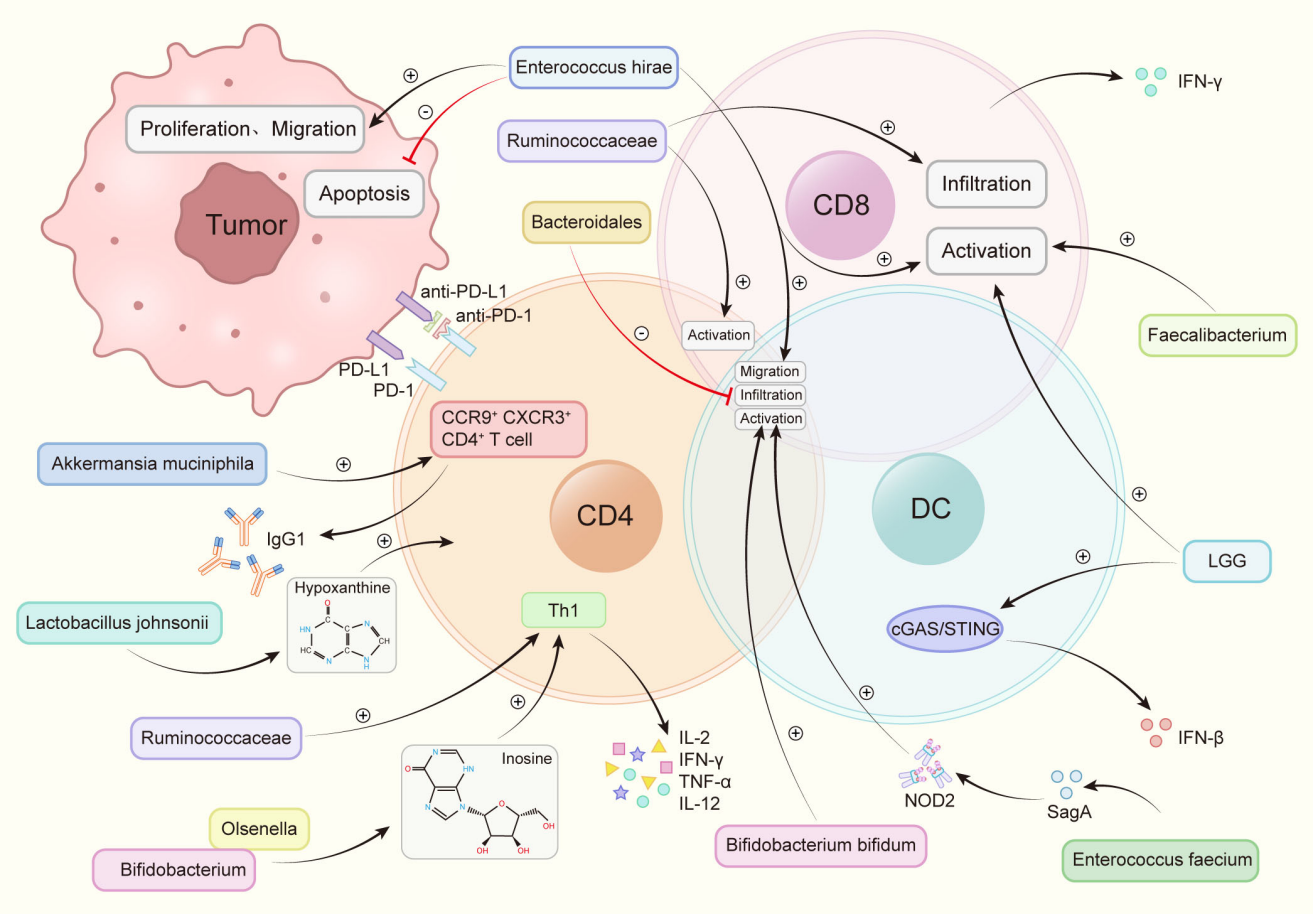
Intestinal flora increases the anti-PD-1 therapeutic effect by regulating immune cells. *Akkermansia muciniphila* accelerated the activity of CXCR3^+^CCR9^+^CD4^+^T cells, produced more IFN-γ. *Bacteroidales* inhibited the infiltration of B cells and T cells. *Bifidobacterium* and *Olsenella* produced metabolites inosine and increase the levels of interleukin-12 (IL-12), interferon γ (IFN-γ), TNF-a and interleukin-2 (IL-2). *Bifidobacterium bifidum* promotes the activation of T cells and B cells. *Enterococcus faecium* releases the metabolite SagA, produces NOD2 active polypeptide, and promotes the activation of B cells, T cells. *Enterococcus hirae* promotes tumor cell apoptosis, weakens the proliferation and migration of tumor cells, promotes the infiltration of T cells, B cells, promotes the activation of CD8^+^ T cells. *Faecalibacterium* promotes the activation of CD8^+^ T cells. *Lactobacillus rhamnosus GG* (*LGG*), induces IFN-β production, promotes CD8^+^ T cell activation via the cGAS/STING pathway in DC. *Lactobacillus johnsonii* enhances the immunotherapy effect through the metabolite hypoxanthine. *Ruminococcaceae* increases the number of effector T cells, induces the activation of effector CD4^+^ and CD8^+^ T cells, increases tumor CD8+ T cell infiltration.

The transfer of fecal microbiota from cancer patients who exhibited a positive response to ICIs into germ-free mice or mice treated with broad-spectrum antibiotics has enhanced the anti-tumor efficacy of anti-PD-1. Conversely, FMT from non-responsive patients did not yield the same improvement (76). At present, through 16s rRNA gene sequencing, nine strains have been identified that substantially enhance the efficacy of anti-PD-1 (**Figure 2**).

##### 2.3.1.1 Bifidobacterium bifidum

The promotion of DC maturation, increased cytokine secretion, and stimulation of the DC-IL-12-Th1-skewing immune response are reported as effects of *Bifidobacterium bifidum*. Moreover, this genus is found to enhance the overall function of DC, fostering the activation and survival of tumor-specific T cells. The Th1-skewing activation mediated by the DC-IL-12 axis contributes to the improvement of the therapeutic response to PD-1 blockade (47). Interferon-γ(IFN-γ) production can be improved by enhancing the biosynthesis of immune-stimulating molecules and metabolites, and oral *bifidobacterium* enhances tumor suppression to the same extent as specific antibody PD-L1 immunotherapy. *Bifidobacterium bifidum* significantly upregulates the secretion levels of IFN-γ and promotes the production of tumor-specific CD8^+^ T cells (47). Activation of CD8^+^ T cells and DCs results in enhanced efficacy of immunotherapy against melanoma, NSCLC, and Renal Cell Carcinoma (RCC) (47, 76, 85). Furthermore, *Bifidobacterium pseudocatenulatum* is found to secrete inosine, amplifying the impact of immunotherapy. The combination of inosine with immunotherapy significantly boosts the anti-tumor activity of T cells across various tumor types (44).

##### 2.3.1.2 *Akkermansia muciniphila*(*AKK*):

A metagenomic examination of fecal samples from cancer patients reveals a robust connection between the clinical effectiveness of ICIs and the prevalence of *AKK* bacteria. Through the facilitation of the recruitment of CCR9^+^CXCR3^+^CD4^+^ T lymphocytes to the tumor tissue in mice, *AKK* bacteria can initiate an antigen-specific T cell response. This process promotes the production of IgG1 antibodies and reinstates the therapeutic efficacy of anti-PD-1 treatment for RCC, melanoma, and NSCLC in an IL-12-dependent manner (76).

##### 2.3.1.3 Enterococcus faecium

*Enterococcus faecium*, expressed NlpC/p60 peptidoglycan hydrolase SagA, can release peptidoglycan fragments, produce Nucleotide-binding oligomerization domain 2 (NOD2) active polypeptides, activate innate immunity, induce a microenvironment conducive to anti-PD-1 immunotherapy, enhance the effectiveness of melanoma immunotherapy (86).

##### 2.3.1.4 Enterococcus hirae

The antitumor mechanism of *Enterococcus hirae* involves inhibiting tumor proliferation, promoting a proinflammatory state, and promoting tumor cell apoptosis. *Enterococcus hirae* can further promote the migration of immune cells to promote anti-tumor effect of immune-targeted drugs. This genus inhibited the proliferation and metastasis of tumor cells like B16F10 melanoma, HT-29 human colon cancer, HepG-2 liver cancer cells (87). Studies have revealed that *Enterococcus hirae* can additionally induce CD8^+^ T cells, a function positively correlated with a good prognosis in HBV-associated hepatocellular carcinoma (HCC) (88).

##### 2.3.1.5 Lactobacillus johnsonii

*Lactobacillus Johnsoni* produces the metabolite hypoxanthine, which then binds to inosine receptors, improving the effectiveness of anti-PD-1 therapy against MC38 colon cancer (44).

##### 2.3.1.6 Olsenella

Much like the situation with *Bifidobacterium pseudocatenulatum*, *Olsenella* amplifies the therapeutic effects by metabolizing inosine. The immunotreatment-induced reduction in intestinal barrier function heightens the systemic transport of inosine, fostering the anti-tumor activation of Th1 cells. This robustly augments the anti-tumor functionality of T cells across diverse tumor types (44). Additionally, the effectiveness of anti-PD-1 therapy in NSCLC is elevated by augmenting the release of IL-12 and IFN-γ (80).

##### 2.3.1.7 *Lactobacillus rhamnosus GG* (*LGG*)

*LGG* heightens the production of tumor-infiltrating DC cells and T cells. When administered in combination with anti-PD-1, *LGG* expedites interferon (IFN) production in DCs, enhancing the generation of anti-tumor CD8^+^ T cells. Consequently, this fosters immune cell activation and augments the immune effectiveness of anti-PD-1 therapy in colorectal cancer(CRC) and melanoma (82).

##### 2.3.1.8 Ruminococcaceae

*Ruminococcaceae* improves the infiltration of CD8^+^ T cells within tumors, induces the differentiation of effector CD4^+^ and CD8^+^ T cells, and enhances the ratio of CD8^+^/FOXP3^+^CD4^+^ in tumor-infiltrating T cells. As a result, this increases the immune efficacy of anti-PD-1 therapy in melanoma and NSCLC (8, 89).

##### 2.3.1.9 Faecalibacterium

*Faecalibacterium* is associated with the increase in CTL concentration in the tumor, subsequently potentiating the immune efficacy of anti-PD-1 immunotherapy on melanoma (8).

The aforementioned flora has all been revealed to activate immune cells through distinct mechanisms of action, thereby enhancing the efficacy of immunotherapy. Individuals who possess these microorganisms in their gut microbiome tend to be more responsive to PD-1 therapy compared to those who do not.

#### 2.3.2 Inhibitory impact of intestinal flora on anti-PD-1 immunotherapy

Certain bacteria tend to accumulate in patients who show lower responsiveness to anti-PD-1 drugs, and these bacteria might directly diminish the effectiveness of PD-1 drugs. For example, *Bacteroidales* restrict the infiltration of lymphocytes and myeloid cells in tumors, dampen antigen presentation activity, and lead to impaired systemic immune and anti-tumor immune responses (8). In melanoma patients with short progression-free survival (PFS), there is a notable abundance of *Bacteroides ovoides* (90). *Bacteroides ovatus*, on the other hand, may influence the immune system by inducing IgA, among other mechanisms (91). A reduction in *Bacteroides ovatus* has been suggested to contribute to the enhancement of the efficacy of anti-PD-1 treatment in CRC (92). Conversely, *Bacteroides thetaiotaomicron*, *Escherichia coli*, *Anaerotruncus colihominis*, *Ruminococcus obeum*, and *Roseburia intestinalis* were found to be enriched in patients resistant to anti-PD-1 immunotherapy (8, 79). However, the specific mechanisms through which these intestinal flora diminish the efficacy of anti-PD-1 therapy remain to be further investigated.

#### 2.3.3 Application of gut microbiota in mouse anti-tumor model.

Currently, several bacteria exhibit anti-tumor effects in mouse tumor models. It has been verified that both endogenous and externally introduced *Akk* significantly impede tumorigenesis in the Lewis lung cancer mouse model (93). Pasteurized *AKK* has been shown to mitigate colitis and colitis-associated CRC by regulating CTLs in a mouse model induced by dextran sulfate sodium (94, 95). Oral administration of *Bifidobacterium* alone enhances tumor control in a mouse model of B16 melanoma, and combined treatment with a PD-L1-specific antibody virtually eliminates tumor growth (47). Furthermore, *B. adolescents* effectively counters Dextran Sulfate Sodium Salt (DSS)-induced chronic colitis by stimulating protective Treg/Th2 responses and remodeling the gut microbiota. Regular administration of *B. adolescents* may enhance treatment outcomes in inflammatory bowel disease (IBD) (96). *Bifidobacterium longum*, *Collinsella aerofaciens*, and *Enterococcus faecium* have also demonstrated efficacy in alleviating colitis in mice, suggesting their potential use as alternative or adjunct therapies for IBD (97). In the MCA205 mouse tumor model, *Enterococcus hirae* is found to increase the intratumoral CD8/Treg ratio and enhance the effectiveness of the anti-cancer immunomodulator cyclophosphamide (CTX) (98). *Lactobacillus johnsonii* inhibited tumor growth in a mouse CRC model (99). Additionally, *LGG* recruits a substantial number of neutrophils and macrophages to the tumor site in a mouse orthotopic Bladder Cancer(BC) tumor model and is being explored as a potential substitute for BCG immunotherapy in the treatment of BC (100).

## 3 Discussion

The gut microbiota assumes a crucial role in regulating tumor responses to cancer immunotherapy (79, 92). Clinical approaches to improving the efficacy of tumor therapy have focused on providing microbial formulations with immune-stimulating properties (FMT and other more defined probiotic strains) or improving immunotherapy outcomes through targeted depletion of immunosuppressive bacteria. FMT can modify the balance between beneficial and detrimental bacteria in the gut microbiome during anti-PD-1 therapy, leading to an enhancement in therapeutic efficacy (92). The role of gut microbes is gaining prominence in the treatment of Non-Small Cell Lung Cancer (NSCLC), RCC, Urothelial Carcinoma (UC), and HCC (76, 101).

Currently, research regarding intestinal flora is primarily challenged by the following complications. Further study on immunomodulatory mechanism could facilitate the precise identification of immune-promoting and immunosuppressive bacterial strains or pathways, which could improve therapeutic efficacy, This may further aid in the avoidance of adverse events such as infections caused by FMT. The symbiotic nature of the microbiome means that it also influences tumor development, suggesting that individuals at high risk of cancer could be screened by the composition of their microbiome, and cancer be prevented by modulating the microbiome of high-risk individuals (102). At present, more accurate detection methods are required for detecting intestinal flora, these could be applied to the detection of intestinal flora in cancer patients to accurately predict the reaction or drug resistance of different intestinal flora to ICI. Similarly, correlation does not imply causation, and therefore, to overcome individual differences and biases, continued clinical trials are required to verify and ultimately achieve the deconvolution of any causal relationship between intestinal flora and immunotherapy effect to improve the effectiveness of ICB cancer treatment.

## Author contributions

XG: Conceptualization, Investigation, Writing – original draft, Writing – review & editing. JJ: Conceptualization, Investigation, Methodology, Supervision, Writing – review & editing.
